# Ezrin Mediates Invasion and Metastasis in Tumorigenesis: A Review

**DOI:** 10.3389/fcell.2020.588801

**Published:** 2020-11-10

**Authors:** Yanan Song, Xiaokun Ma, Miao Zhang, Menghan Wang, Guoyu Wang, Ying Ye, Wei Xia

**Affiliations:** ^1^Central Laboratory, The Seventh People’s Hospital of Shanghai University of Traditional Chinese Medicine, Shanghai, China; ^2^Department of Nuclear Medicine, The Seventh People’s Hospital of Shanghai University of Traditional Chinese Medicine, Shanghai, China

**Keywords:** Ezrin, cancer, migration, invasion, metastasis

## Abstract

Ezrin, as encoded by the EZR gene, is a member of the Ezrin/Radixin/Moesin (ERM) family. The ERM family includes three highly related actin filament binding proteins, Ezrin, Radixin, and Moesin. These three members share similar structural properties containing an N-terminal domain named FERM, a central helical linker region, and a C-terminal domain that mediates the interaction with F-actin. Ezrin protein is highly regulated through the conformational change between a closed, inactivate form and an open, active form. As a membrane-cytoskeleton linker protein, Ezrin facilitates numerous signal transductions in tumorigenesis and mediates diverse essential functions through interactions with a variety of growth factor receptors and adhesion molecules. Emerging evidence has demonstrated that Ezrin is an oncogene protein, as high levels of Ezrin are associated with metastatic behavior in various types of cancer. The diverse functions attributed to Ezrin and the understanding of how Ezrin drives the deadly process of metastasis are complex and often controversial. Here by reviewing recent findings across a wide spectrum of cancer types we will highlight the structures, protein interactions and oncogenic roles of Ezrin as well as the emerging therapeutic agents targeting Ezrin. This review provides a comprehensive framework to guide future studies of Ezrin and other ERM proteins in basic and clinical studies.

## Introduction

Cancer is one of the most debilitating diseases worldwide. The molecular mechanisms of carcinogenesis provide essential implications for potential prevention and treatment of cancers. Extensive studies have been conducted on tumor invasion and metastasis, and multi-step processes have been described. Previous research has shown that cell adhesion, migration, and morphogenesis regulate tumor invasion and metastasis (Janiszewska et al., 2020). However, adhesion complexes, reorganization of the cytoskeleton, and their underlying molecular mechanisms are still poorly defined.

The Ezrin/Radixin/Moesin (ERM) family proteins regulate cell networks through linking actin cytoskeleton to the cell membranes (Kong et al., 2013). ERM family members, actin cytoskeleton and the cell membranes form highly dynamic domains including lamellipodia and filopodia (Baumgartner et al., 2006). ERM family proteins switch between a closed (inactive) and an open (active) conformation to work with their interacting partners, which is tightly regulated by phosphorylation through different kinases (Matsui et al., 1998).

Ezrin, a member of ERM family, is phosphorylated by threonine and tyrosine kinases (Srivastava et al., 2005). Ezrin is a highly conserved protein through evolution, suggesting the same regulatory mechanisms between organisms (Fouassier et al., 2000). Ezrin mediates signal transduction, coordinates dynamic cellular processes, and acts through cytoskeletal reorganization (Bretscher et al., 2002). Genetic ablation experiments have confirmed the pleiotropic effects of Ezrin including cell polarity, adhesion, and invasion (Clucas and Valderrama, 2014). Ezrin controls signaling transduction by interacting with adhesion molecules and various growth factor receptors (Khanna et al., 2004; Auvinen et al., 2013). In this review, we focus on Ezrin’s distinct roles in tumor growth, metastasis, and morphogenesis in cancer biology, because increased Ezrin expression is correlated with poor prognoses in various cancers. In addition, we address Ezrin’s signaling pathways in cancer development and prognosis.

## Structure, Function, and Signaling Pathways

### Structure and Activation

**FIGURE 1:**
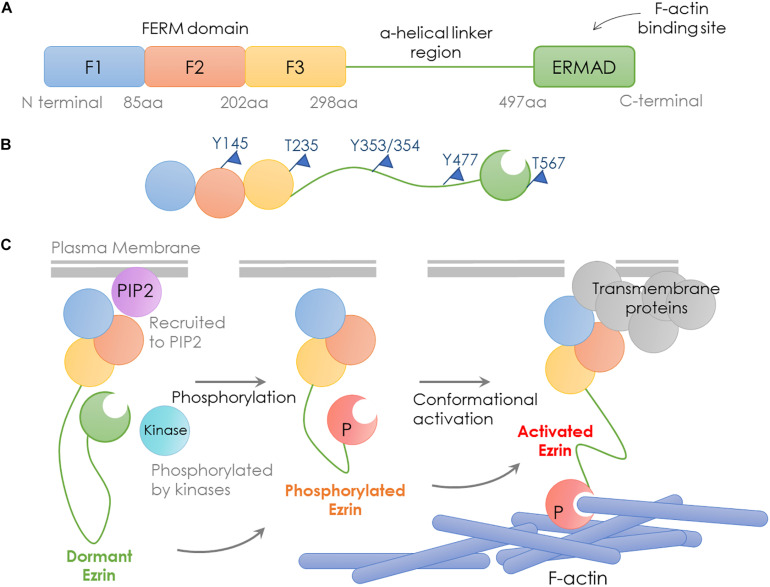
Structure and activation process of Ezrin. Schematic representation of domain structure, activation states and binding partners of Ezrin protein. **(A)** Domain structure of Ezrin includes the N-terminal FERM domain (band 4.1 protein, Ezrin, Radixin, Moesin), the central α-helical linker region and the C-terminal ERM-associated domain (C-ERMAD, green). The FERM domain comprises three subdomains, F1, F2, and F3 (blue, red, and yellow) and C-ERMAD contains the F-actin-binding site. **(B)** The putative open state of Ezrin protein and its phosphorylation sites. **(C)** Various states and binding partners of Ezrin protein. (1) Ezrin is phosphorylated at several sites (e.g., T567 in Ezrin, T564 in Radixin and T558 in Moesin); (2) Ezrin is recruited to PIP2; (3) Activated Ezrin monomer (or head-to-tail dimer) binds with F-actin; (4) Ezrin binds with transmembrane receptors such as CD43/44, ICAM1/2 and NHE-1.

Encoded by the EZR gene that locates at chromosome 6q25.2-q26, the Ezrin protein is the most studied member of the Ezrin/Radixin/Moesin (ERM) family, containing an FERM domain (band 4.1 protein, Ezrin, Radixin, Moesin), a central helical linker region and an ERM-associated domain (Figure 1A; Yin et al., 2018).

The conformational change in Ezrin determines its activity. When the NH_2_- and COOH-terminal bind to each other, full length Ezrin is in a closed inactive form. The abolition of the intramolecular head-to-tail interaction is required to expose the actin binding sites, since the F-actin binding site at the C-terminal domain is normally masked in the full length Ezrin (Gary and Bretscher, 1995; Roy et al., 1997). Therefore, full length Ezrin is inactive and cannot interact with actin (Fehon et al., 2010). Activated Ezrin directly binds F-actin through a C-terminal domain (Bretscher et al., 1997). Direct binding of F- and G-actin occurs at the Ezrin N-terminal domain between residues 281 and 333 (Roy et al., 1997).

Ezrin dimers and higher oligomers present as inactive, and monomers are considered active (Gautreau et al., 2000). Multiple sites in ERM family proteins can be phosphorylated by several kinases and exhibit various biological functions (Table 1). Specifically, phosphorylation of the C-terminal threonine residue (Thr567) is the key step to activate Ezrin, which allows the actin filament binding domains to interact with other proteins and break head-to-tail associations (Figure 1B; Matsui et al., 1998). The threonine phosphorylation is a Rho-dependent activation of Ezrin (Chen et al., 2011). Besides threonine phosphorylation, tyrosine phosphorylation in Ezrin (Tyr353) is linked to p85 interaction and Akt overexpression (Cui et al., 2010). Together with Thr567, Tyr353 regulates Ezrin’s transition to its active form (Jin et al., 2014). Phosphorylation contributes to a plasma membrane mediated transition from Ezrin oligomers and monomers *in vivo* (Gautreau et al., 2000). The regulation of Ezrin phosphorylation is complex (McClatchey, 2003). It is also reported that *in vivo* phosphorylation of Ezrin is required in its binding and recruiting to the plasma membrane phospholipid phosphatidylinositol 4,5-bisphosphate (PIP2) (Fievet et al., 2004; Hao et al., 2009). Ezrin interacts with other kinases, including myotonic dystrophy kinase-related Cdc42-binding kinase (Nakamura et al., 2000). Additionally, Src kinases and RhoA/Rho kinase activities are required for ERM activation, a key step in the growth of cone filopodia for axon outgrowth (Antoine-Bertrand et al., 2011). Interestingly, Ezrin mediates focal adhesion kinase activation independently from external stimuli (Poullet et al., 2001). Although phosphorylation of Ezrin is the most studied post-translational regulation, the biological effects of the phosphorylation sites are largely unexplored (Michie et al., 2019).

**TABLE 1:** Ezrin phosphorylation sites and kinases.

Phosphorylation sites	Kinases	Functions	References
Y145	Hepatocyte growth factor (HGF) receptor, Lck	Activate Ezrin, enhances migration and tubulogenesis; T cell activation	Crepaldi et al., 1997; Autero et al., 2003
T235	Cyclin-dependent kinase 5	Induce the release of Rho GDP dissociation inhibitor, increase interaction with Rac1	Yang and Hinds, 2006
Y353/354	Hepatocyte growth factor receptor	Activate Ezrin, enhances migration and tubulogenesis; Responsible for the interaction with p85, required for PI3-kinase and Akt activation mediated cell survival; Nuclear localization	Crepaldi et al., 1997; Gautreau et al., 1999; Di Cristofano et al., 2010
Y477	Src	Not related to head-to-tail conformational opening, is associated with kelch-repeat superfamily protein; Regulates invasion and metastasis	Heiska and Carpen, 2005; Mak et al., 2012
T567 in Ezrin (T564 in Radixin and T558 in Moesin)	Rho-associated kinase, protein kinase B2/Akt2, atypical protein kinase C-iota (aPKCι), mammalian Sterile 20 (Ste20)-like kinase-4 (Mst4), lymphocyte-oriented kinase (LOK) and Ste20-like kinase (SLK)	Interferes with the intermolecular head-to-tail association, activates Ezrin and is positively associated with invasive growth	Matsui et al., 1998; Shiue et al., 2005; Wald et al., 2008; Gloerich et al., 2012; Viswanatha et al., 2012; Antelmi et al., 2013

As discussed earlier, unphosphorylated/inactive Ezrin remains in a folded conformation through head-to-tail interaction, masking binding sites for other molecules. Phosphorylation on the conserved threonine residue T567 causes conformational changes, unmasking binding sites (Matsui et al., 1998; Figure 1B). T567 keeps Ezrin open and active, and prolongs its lifetime (Prag et al., 2007). Phosphorylated Ezrin is involved in fiber formation, adhesion, and migration (Shiue et al., 2005; Viswanatha et al., 2012; Antelmi et al., 2013). To regulate cytoskeleton dynamics, the EMR family proteins directly interact with actin filaments to link the cytoskeleton to the plasma membrane (Figure 1C). The FERM domain is essential for Ezrin’s binding partners including intercellular adhesion molecules (ICAMs) 1–3, CD43/44, and NHE-1 (Denker et al., 2000; Ivetic and Ridley, 2004). Importantly, anti-metastatic small molecules NSC30587 and NSC668394 were identified that directly target Ezrin T567 phosphorylation and inhibit Ezrin’s actin binding (Bulut et al., 2012). Therefore, targeting Ezrin phosphorylation and actin binding activity provides a new therapeutic direction for clinical cancer interventions.

### Ezrin’s Function

#### Physiological Roles

**FIGURE 2:**
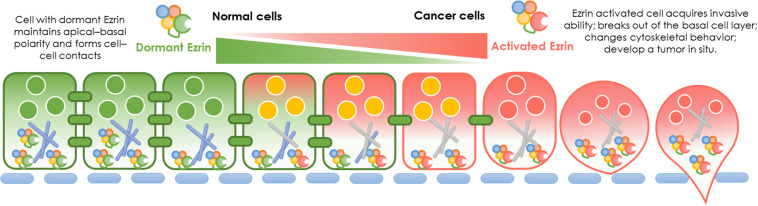
Ezrin change between normal cells and cancer cells. Physiological function and pathological effect of Ezrin protein in normal cells versus cancerous cells. Under physiological conditions, Ezrin proteins arrange the cytoskeleton of epithelial cells, mediate signaling pathways to generate an apical–basal polarity, normal cell morphology and consistent cell–cell contacts. Under pathological conditions, Ezrin proteins are upregulated and activated to promote cancer progression and metastasis in various types of epithelial cancers (breast, lung, and prostate). The relative membrane localization of Ezrin protein is increased, cell–cell contacts disrupted and therefore facilitates the process of cancer invasion.

In normal cells, Ezrin protein is known to contribute to epithelial morphogenesis, adhesion, and migration (Figure 2). Under physiological conditions, Ezrin maintains the cytoskeleton and normal shapes of epithelial cells. It mediates signaling pathways to maintain an apical–basal cellular polarity, as well as normal cell morphology, and binds to actin filaments to keep consistent cell–cell contact. In cancer cells, Ezrin is significantly activated, phosphorylated, and elevated, enhancing cancer cells’ invasive abilities (Figure 2).

#### Cancer-Promoting Roles

During cancer development, the relative membrane localization of Ezrin proteins is increased and cell–cell contact is disrupted. Therefore, the activation of Ezrin proteins facilitates the process of tumor progression and invasion. The effect of enhanced Ezrin proteins in cancer metastasis takes different forms in various cancers. For example, in osteosarcoma, Ezrin allows metastatic tumor cells to overcome a number of stresses as cells from the primary lesion are able to break loose and effectively initiate the growth of secondary lesions by generating additional ATP from a variety of sources (Ren and Khanna, 2014; Zhang et al., 2014). In pancreatic cancer cells, Ezrin translocates into plasma membranes, binds to increasing amounts of cortactin, and formes a highly ordered structure called a podosomal rosette, which enables epithelial cancer cells to adhere to the underlying substrate and modify their cytoskeletal behaviors (Kocher et al., 2009). Moreover, Ezrin is responsible for cellular polarization in pancreatic cancer associated macrophages (Chang et al., 2020). Enriched Ezrin expression has been detected in salivary gland carcinomas, which was significantly correlated with the levels of other cancerous molecular markers such as Ki67, HER2, p53, male sex, high-grade histopathology, and distant tumor metastasis (Hashimoto et al., 2017). Additionally, in lung cancer cells, activated Ezrin facilitates mechanical transduction from the cytoskeleton to the membrane and regulates the malignant process in a tension-dependent manner (Zhang et al., 2019).

### Ezrin’s Signaling Pathways

#### Ezrin Interacts With Multiple Signals Through Spatial and Temporal Regulation

Activated Ezrin regulates key events and interacts with different proteins in a variety of cancer types. The precise spatial and temporal activation of Rho GTPases establishes cell polarity and morphology (Haga and Ridley, 2016). The antagonistic relationships between different Rho GTPases regulate migration and adhesion, consistent with their opposing effects on ERM family proteins (Ivetic and Ridley, 2004). Ezrin recruits Cdc42, and the conformational active (phosphorylated) Ezrin brings Rho/Cdc42 specific guanine nucleotide exchange factor Dbl to the membrane. A precise spatial Dbl activated Cdc42 is crucial for directional cell migration in breast cancer cells (Prag et al., 2007). Dysfunction and loss of cell adhesion has been recognized as a pro-tumorigenic step, which enables the cancer cell to migrate and metastasize.

#### Ezrin Mediates E-Cadherin-Catenin Complex Maintenance

The E-cadherin-catenin adhesion complex maintains tissue architecture and is critical for intercellular adhesiveness. Ezrin suppression promotes the expression of E-cadherin and β-catenin. Both E-cadherin and β-catenin play a key role in epithelial cell adhesion. Co-precipitation experiments suggests Ezrin associates with E-cadherin and β-catenin (Hiscox and Jiang, 1999). The modulation between Ezrin and E-cadherin is mediated by IL-1β and TGF-β1, suggesting that cytokine regulation in tumor invasion is governed by alteration in cell-cell interactions (Karmakar and Das, 2004).

#### Other Signaling Molecules

Ezrin mediates cell growth and survival through Akt signaling, but not the mitogen-activated protein kinase (MAPK) pathway in certain cancers, which is essential for cancer proliferation, invasion, migration and survival (Krishnan et al., 2006; Hu et al., 2016; Quan et al., 2019). Ezrin is correlated with poor prognoses in these cancer patients (Quan et al., 2019). In addition, Ezrin is associated with the p85 subunit, activating phosphatidylinositol 3-kinase (PI3K)/Akt in regulating tumorigenesis, metastasis, cell survival, and invasion in epithelial cells (Gautreau et al., 1999; Cui et al., 2010).

## Ezrin’s Roles in Cancers

**FIGURE 3:**
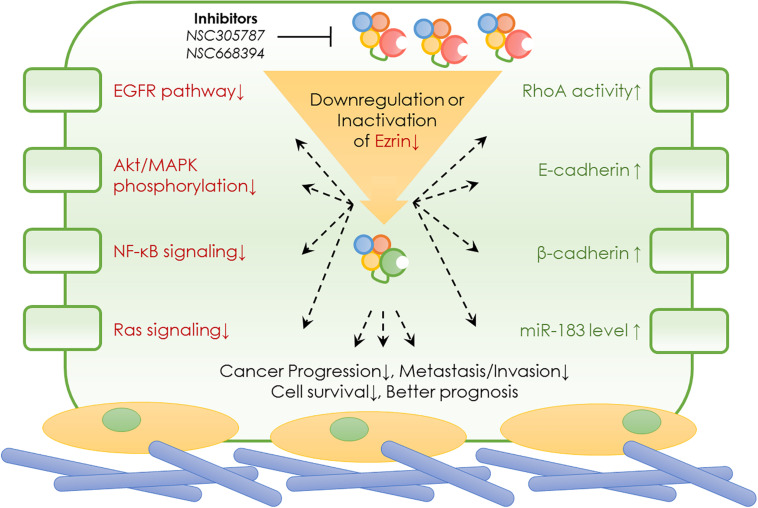
Ezrin mediated signaling pathways and its pharmacological inhibitors.

As an important member of the ERM family of proteins, Ezrin has been well studied in many cellular events. As summarized earlier, Ezrin plays a vital role in molecular signaling, including cell proliferation, cell polarity establishment, cell motility, and cell adhesion (Ren and Khanna, 2014; Kawaguchi et al., 2017). Since these processes are crucial in invasion, and metastasis in a variety of solid tumors, the pathophysiological roles of Ezrin protein were extensively studied and discussed (Kawaguchi et al., 2017). Although Ezrin is known associated with poor prognosis in several cancers, the predictive value of Ezrin and its relationships with clinicopathological features or prognostic parameters remain controversial (Cihan, 2018). It is interesting to note that the Ezrin expression was associated with bad prognosis in a cancer type-specific manner (Li et al., 2015). In few cases such as bladder cancer, higher Ezrin expression indicates better prognosis rather than worse. In order to draw a most recent conclusion from the up-to-date work, below we summarized the specific roles of Ezrin in various cancers, highlighting the special signaling cascades and pathophysiological roles (Figure 3 and Table 2).

**TABLE 2:** Selective Ezrin interacting proteins in various cancers.

Cancers	Interacting proteins	Roles	References
Lung cancer	EGFR, ROCK1/2, RhoA, Akt signaling	Promotes cell migration and invasion	Lee et al., 2012; Li et al., 2012; Chen et al., 2013; Hata et al., 2016; Saygideger-Kont et al., 2016; Moodley et al., 2020
Breast cancer and ovarian cancer	miR-183, Akt signaling	Promotes cancer progression and enhances metastasis	Lowery et al., 2010; Horwitz et al., 2016; Ghaffari et al., 2019; Li et al., 2019
Cervical cancer	LGALS1, Galectin-3, E-cadherin, MAPK JNK/p38 and Akt/ERK1/2 signaling	Controls cell adhesion and enhances invasion. Serves as the cervical cancer marker for non-invasive detection	Elliott et al., 2005; Saito et al., 2013; Kong et al., 2016; Fadiel et al., 2017; Li et al., 2017; Zacapala-Gomez et al., 2018; Chetry et al., 2020
Gastric cancer	miR-183	Antagonizes mi-183 actions, and is correlated with tumor size, invasion lymph node and metastasis	Li et al., 2011; Cao et al., 2014
Osteosarcoma	miR-183, Akt and MAPK, Akt/mTOR	Promotes tumor metastasis. High expression is correlated with poor prognosis	Khanna et al., 2004; Krishnan et al., 2006
Hepatocellular cancer	Rho kinase, cytokeratin 19	Positive expression is correlated with a smaller tumor size and higher frequency of tumor dedifferentiation	Okamura et al., 2008; Yeh et al., 2009

### Ezrin in Lung Cancer

Highly expressed Ezrin has been detected in lung cancer cell lines and primary lung cancer tissues. Ezrin has been found to be primarily distributed in the cytoplasm of lung cancerous tissue and metastatic foci (Wang et al., 2008; Lee et al., 2012; Zhang et al., 2012; Jin et al., 2014; Kolegova et al., 2020). Ezrin expression correlates to the degree of lymphatic metastasis, malignant phenotype, and advanced TNM staging of lung cancer patients significantly (Lee et al., 2012; Li et al., 2012). Not surprisingly, downregulation of Ezrin was observed to reverse these aggressive biological behaviors (Chen et al., 2012; Lee et al., 2012). The underling molecular mechanism of Ezrin activation in lung cancer involves Ezrin modifications (such as phosphorylation and S-nitrosylation), epidermal growth factor receptor (EGFR), and EGFR-mediated signaling pathways in non-small cell lung cancer (NSCLC) cells (Saygideger-Kont et al., 2016; Zhang et al., 2019). Downregulation of Ezrin in lung cancer cells has resulted in actin cytoskeleton rearrangements, reduced EGFR activity and phosphorylation levels of downstream signaling pathways, as well as a substantial reduction in cell migration and invasion (Chen et al., 2013; Saygideger-Kont et al., 2016). Ezrin also mediates downstream signaling pathways, including the activation of RhoA-GTPase and the signaling of ROCK1/2 and Akt in lung adenocarcinoma (Hata et al., 2016; Moodley et al., 2020). Interestingly, Ezrin serum levels were negatively correlated with serum IL-13 levels (which are believed to play an important role in lung function) (Jia et al., 2019).

### Ezrin, Breast Carcinoma, and Ovarian Carcinoma

Similar to observations in other carcinomas, Ezrin is elevated in breast carcinoma and ovarian carcinoma. Ezrin plays a critical role in extracellular matrix remodeling and tumor dissemination in a 3-dimensional model (Horwitz et al., 2016). Since both breast and ovarian carcinomas exhibit a similar ability to disseminate due to malignant effusion formation, the significant increase of Ezrin serves as a future therapeutic intervention target. Ezrin mediates cell migration and invasion in lung and breast cancers that can be inhibited by the overexpression of miR-183 (Lowery et al., 2010). Ezrin promotes breast cancer progression and enhances metastasis through Akt signaling (Li et al., 2019). Elevated Ezrin expression increases the risk of relapse in node-positive and high-risk node-negative breast cancer patients. Pharmacological inhibition of Ezrin has significantly reduced cancer cell migration and invasion into the lymph nodes and lungs *in vivo* in real time (Ghaffari et al., 2019).

### Ezrin in Cervical Cancer

Cervical cancer is the fourth most common cause of cancer-causing death in women. Cervical cancer originates from an epithelial neoplastic transformation in the uterine cervix. Cervical cancer is generally caused by an infection of the human papillomavirus (HPV) (Saavedra et al., 2012). As a migration-related protein, Ezrin is upregulated in cervical cancer (Zacapala-Gomez et al., 2018; Chetry et al., 2020) and its expression level is associated with advanced metastasis and poor prognosis. Specifically, Ezrin is increased in cervical cancer cells (SiHa and C33A) when Galectin-1 (LGALS1) is overexpressed. Ezrin expression is significantly suppressed when LGALS1 is downregulated. LGALS1 belongs to the carbohydrate-binding protein family and exhibits a high affinity for β-galactoside-containing glycol-conjugates (Chetry et al., 2020). Although multiple signaling pathways linked to LGALS1 have been reported, the underlying mechanisms of how LGALS1 affects Ezrin levels have not been fully elucidated. LGALS1 may interact with Ezrin through the MAPK, JNK/p38, and Akt/ERK1/2 pathways in the regulation of invasion and migration (Elliott et al., 2005; Chetry et al., 2020). Another lectin family member, Galectin-3, is also overexpressed along with Ezrin in cervical cancer and both are predictors of poor prognosis in cervical cancer patients (Li et al., 2017). Ezrin down-regulation induces Akt phosphorylation, and Ezrin regulates both epithelial-mesenchymal transitions and metastasis in cervical cancer (Kong et al., 2016). Ezrin promotes cell proliferation through phosphorylation on residue Y145 (Gautreau et al., 1999; Saito et al., 2013), cell mobility, and migration in cervical cancer cells (Kong et al., 2016).

Ezrin plays a key role in cervical cancer invasion and is a potential prognostic immunomarker. Interestingly, Ezrin expression is correlated with HPV associated lesions (Auvinen et al., 2013; Zacapala-Gomez et al., 2018), suggesting that Ezrin can be used to distinguish between transient and persistent HPV integration. More than 80% of cervical cancer samples exhibit high Ezrin expression and a decrease in E-cadherin levels, which can be detected using immunohistochemistry and cervical smears (Zacapala-Gomez et al., 2018). Consistent with this study, the overlapping of Ezrin and estrogen receptor expression during cervical carcinogenesis raises the possibility that Ezrin is associated with the penetration of the basement membrane (Fadiel et al., 2017). Therefore, Ezrin controls adhesion and the invasiveness of cancer cells through the interactions between cell adhesion molecules, suggesting a role in developing cervical neoplasia and cancer. Due to the high expression of Ezrin, a non-invasive testing method can serve as a milestone for cervical cancer detection, which is crucial for early treatment and a better prognosis in patients with squamous intraepithelial lesions. In addition to cervical cancer, enhanced Ezrin expression is a new, independent prognostic marker in endometrioid carcinoma and is correlated with endometrioid carcinoma stages (FIGO) (Kobel et al., 2006).

### Ezrin and Gastric Cancer

Gastric cancer is the second most prevalent cause of cancer death. The Ezrin protein is up-regulated in gastric cancer lesions. Ezrin expression is correlated with tumor size, tumor location, lymph node invasion and metastasis, and shortened survival in stages I, II, and III (Li et al., 2011). Specifically, Ezrin can be used as an early diagnostic marker and to predict later metastasis in gastric cancer using meta-analysis (Jin et al., 2012; Liang et al., 2017). Decreased miR-183 and elevated Ezrin have been reported in gastric cancer cells and tissues. The 3′UTR region of Ezrin’s mRNA is a direct target of miR-183. miR-183 antagonizes Ezrin and acts as a tumor suppressor in gastric cancer (Cao et al., 2014).

### Ezrin and Osteosarcoma

Dysregulation of miR-183 through Ezrin targeting promotes osteosarcoma tumor metastasis. Ezrin is required for metastasis in osteosarcoma and its high expression is associated with poor outcomes in pediatric osteosarcoma patients. Khanna et al. (2004) has reported that suppression of Ezrin deceases Akt and MAPK phosphorylation, but Ezrin induced metastatic survival is mediated partially by MAPK instead of Akt. Interestingly, Khanna et al. (2004) have reported that Ezrin-mediated growth and survival in Ewing sarcoma is dependent on Akt/mTOR, but not MAPK (Krishnan et al., 2006), suggesting that Ezrin acts through different signaling pathways in different cancers. Ezrin’s roles in Ewing sarcoma are distinct from its roles in other sarcomas. A majority of Ewing sarcoma samples express Ezrin, but the intensity and expression pattern of Ezrin is not correlated with clinical characteristics. In contrast to Ezrin’s roles in promoting carcinogenesis, Ewing sarcoma patients with high Ezrin intensity had a superior 5-year event-free survival compared to patients with low or no Ezrin expression (Cash et al., 2017). However, other factors, including diagnosis time, tumor size, therapeutic treatment, and larger sample size should be considered to validate the correlation between Ezrin and Ewing sarcoma clinical outcomes.

### Ezrin and Hepatocellular Cancer

As discussed earlier, Ezrin phosphorylation regulation contributes to Ezrin’s molecular plasticity. Hyperphosphorylation at the C-terminal threonine residue (T567) is significantly correlated with an invasive clinical hepatocellular carcinoma (HCC) (Chen et al., 2011). Therefore, blocking Rho kinase-mediated Ezrin phosphorylation can inhibit liver tumor metastasis. Ezrin staining in HCC is dramatically associated with cytokeratin 19 expression. Ezrin-positive patients had increased serum α-fetoprotein, shortened recurrence-free periods, and shortened overall survival (Okamura et al., 2008). Ezrin is expressed in hepatic progenitor cells, and some cases of HCC are derived from hepatic progenitor cells. Ezrin overexpression is involved in the dedifferentiation and invasion of hepatitis B virus-associated HCC (HBV-HCC). Surprisingly, patients with positive Ezrin expression had smaller tumor sizes and a higher frequency of tumor dedifferentiation and vascular invasion. Ezrin expression is independently associated with tumor size, poor differentiation, and vascular invasion in HBV-HCC (Yeh et al., 2009).

### Ezrin and Bladder Cancers

Inconsistent with most of cancers mentioned above, membranous expression of Ezrin is significantly lower in high grade bladder cancer and significantly associated disease-specific overall survival (Palou et al., 2009; Athanasopoulou et al., 2013). Ezrin is an independent predictor of muscularis propria invasion and increased progression. Unlike its role in other cancers, reduced membranous Ezrin expression is related with unfavorable clinicopathological characteristics and an impaired survival (Andersson et al., 2014). Although these reports collectively suggested the prognostic value of Ezrin in bladder cancer, its immunohistochemical expression level failed to predict therapy effect (Malmstrom et al., 2017).

### Ezrin and Other Cancers

Ezrin expression negatively correlated with renal cell carcinoma (RCC) metastasis, and the inhibition of Ezrin expression suppressed the invasive abilities of RCC cells (Yu et al., 2015). Using immunohistochemical approaches, Ezrin reactivity was observed mainly in conventional, papillary, and mucinous tubular spindle cell carcinoma subtypes of RCC, suggesting that the Ezrin protein might be beneficial as an additional diagnostic marker in the differential diagnosis of RCC subtypes (Tuna et al., 2009).

In colorectal cancer (CRC), Ezrin binds with a cell-neural adhesion molecule (L1CAM) and mediates the phosphorylation of NF-κB as well as the activation of NF-κB signaling (Gavert et al., 2010). It has also been reported that increased expression of Ezrin (phosphorylated on T567) was seen in liver metastasis in an insulin-like growth factor type 1 receptor (IGF1R)-dependent CRC xenograft model as compared to primary CRC. The Ezrin protein induces CRC cell survival through the modulation of apoptosis protein inhibitor XIAP, which was dependent on T567 (Leiphrakpam et al., 2014). Several studies have confirmed that Ezrin may serve as a promising biomarker in estimating the prognosis, outcome, and differential status of CRC patients (Patara et al., 2011; Lin and Chen, 2013; Fathi et al., 2017; Slik et al., 2017; Aikawa et al., 2019).

In glioblastoma, Ezrin interacts with and delocalizes the cytoskeletal-related protein neurofibromatosis type 2 (NF2), which carries out opposite activities in tumor growth (Morales et al., 2010). Notability, Ezrin, in a complex with NF2, enhances glioblastoma growth independent of its molecular conformation or subcellular localization. Using medulloblastoma cell lines and athymic mice as models, a study reported that Ezrin is localized to filopodia in medulloblastoma cells and promotes filopodia formation as well as *in vitro* invasion in medulloblastoma (Osawa et al., 2009).

In primary melanomas of the skin and metastatic tumors, Ezrin expression correlates with tumor progression and suggests worsening clinical disease behaviors. The molecular mechanism involves molecules related to metastatic functions such as CD44, merlin, and Ras signaling (Ilmonen et al., 2005; Federici et al., 2009; Riecken et al., 2016). Consistent with findings in osteosarcoma, Ezrin was found to be highly expressed in pancreatic cancer tissues and to positively regulate cell proliferation and invasion through the activation of the Akt/mTOR pathway (Meng et al., 2010; Quan et al., 2019; Chang et al., 2020). Ezrin and Rho-A expressions in squamous cell carcinoma suggest a cooperative participation of these proteins in cell movement and invasion (Assao et al., 2017). A tumorigenic role of Ezrin in skin cancer has also been demonstrated using immunohistochemical staining specimens from epithelial skin tumors, together with squamous carcinoma cell lines (Abdou et al., 2011; Wu et al., 2011). A similar result was seen in nasopharyngeal carcinoma as phosphorylated Ezrin expression was dependent on increased Rho kinase and protein kinase C activity (Tang et al., 2011). The oncogenic role of Ezrin is not limited to solid tumors as it has also been seen in blood cancers, such as diffuse large B-cell lymphoma, where the knockdown of Ezrin attenuated chemotherapy resistance (Pore et al., 2015; Sun et al., 2018).

## Ezrin as a Pharmacological Target

The above sections are not meant to recap all the latest important findings in Ezrin research but rather to provide an overview of the evidence showing the oncogenic roles and prognostic value of Ezrin in a wide range of cancer types. One of the questions that remain to be answered is what the clinical implication of Ezrin is. As described above, high levels of Ezrin are observed in many cancers with lung metastasis, indicating poor survival and bad prognoses. Ezrin as an essential prognosis predictor of various cancers has been demonstrated to be a key modulator of tumor metastasis. All the existing studies, taken together, highlighted the fact that Ezrin may serve as a potential therapeutic target in cancer (Hoskin et al., 2019). This prompts the next question whether or not pharmacological regulators with a high affinity to Ezrin would exhibit encouraging results for cancer treatment. Despite various downstream pathways (Figure 3) of Ezrin been identified in cancers, it is expected that identification of small molecule inhibitors of Ezrin would lead to the discovery of anti-metastatic and anti-invasion drugs.

### Small Molecular Inhibitors

Over the past decade, many studies have attempted to develop targeted cancer treatment strategies using small molecule inhibitors of Ezrin (Table 3). For the first time, Bulut et al. (2012) identified two compounds (NSC305787 and NSC668394) from small molecule libraries, which can directly bind to Ezrin, reduce phosphorylation on T567 and block its functional activity. These two inhibitors effectively reduced tumor metastasis in lung cancer and osteosarcoma (Celik et al., 2015, 2016). Following that, more and more studies attempted to extend the anti-metastatic activity of these two small molecule inhibitors in other cancers. Surprisingly, although Ezrin showed widely pro-metastatic capacity in many cancers, the anti-metastatic effect of its inhibitors was only seen in a few cancer types (Table 3). To date, NSC305787 and NSC668394 are undergoing investigation through animal models but not yet included in any clinical trials.

**TABLE 3:** Pharmacological inhibitors and activators targeting Ezrin.

Name	Target site and modification	Experiment models	References
*Inhibitor*			
NSC668394 and NSC305787	Inhibition of T567 phosphorylation	Zebrafish, osteosarcoma cell culture, Xenopus embryonic development, mouse lung organ culture and *in vivo* lung metastasis models	Bulut et al., 2012
Compounds 21k and 21m, as analogs of NSC668394	Inhibition of T567 phosphorylation	*In vitro* binding assays	Paige et al., 2014
NSC668394 and NSC305787	Inhibition of T567 phosphorylation	Mouse lung metastasis cell culture model	Celik et al., 2015, 2016
NSC668394, drug-like compounds MMV020549 and MMV666069	Inhibition of T567 phosphorylation	Zebrafish, osteosarcoma cell culture, and Xenopus embryonic development models	Celik et al., 2015
NSC668394	Inhibition of T567 phosphorylation	Diffuse large B-cell lymphoma cell line and tumor Xenografts mice models	Pore et al., 2015
NSC668394	Inhibition of T567 phosphorylation	Tumor-bearing lymphatic reporter mice model	Ghaffari et al., 2019
NSC305787	Inhibition of T567 phosphorylation	Lung cancer cell model	Moodley et al., 2020
NSC668394	Inhibition of T567 phosphorylation	Japanese encephalitis virus mouse infection model	Liu et al., 2020
*Activator*			
Cyclin-dependent kinase 5, CDK5	Activation of T235 phosphorylation	RB-transfected osteosarcoma cell model	Yang and Hinds, 2003

### Activator

Because of the oncogenic role of Ezrin, studies investigating Ezrin activator are rare. Ezrin activation has been linked to CDK5 in the senescent phenotype as CDK5 is able to activate Ezrin by phosphorylating T235 of Ezrin (Yang and Hinds, 2003). Interestingly, CDK5 mediated activation of Ezrin prevents the intermolecular interactions with/within cell membranes and cooperative with phosphorylation of another site T567, allowing Ezrin to participate in cytoskeleton-related signaling.

## Conclusion and Future Direction

In the literature, the oncogenic roles of Ezrin were intensively studied but there are a limited number of studies investigating the predictive performance of Ezrin expression level. In this review, we summarized not only the oncogenic roles of Ezrin but also its pathophysiological roles and potential pharmacological regulators in a wide range of cancer types. Our understanding of Ezrin as a potential drug target is strongly influenced by the idea that Ezrin is commonly proved to promote tumor metastasis and predicts poor prognosis in different types of cancers. Therefore, direct inactivation of Ezrin by the small molecule inhibitors should provide a new strategy for metastatic treatment in many cancers. While this hypothesis is indeed supported by a few lines of evidence in a couple of cancer types such as lung cancer, this rule seems failed to expand in many other cancer types.

Many fundamental questions in the roles of Ezrin remain to be answered. From this work, some basic understanding of Ezrin protein may be challenged. For example, the expression level of Ezrin in bladder cancer is reduced while it is commonly up-regulated in many other cancer types. The predictive value of Ezrin in bladder cancer is also found opposite to the other cancer types. Given metastasis is a complicated process that involves many steps that are poorly understood at this time, some of which may include tissue type-specific mechanism involving Ezrin. This mechanism may not be shared within ERM family proteins as the other ERM protein Mosin was found enriched in bladder cancer and consistent with its oncogenic role in invasion process. Future work is needed to uncover new pharmacological inhibitors and to explore the *in vivo* activity of the existing small molecule inhibitors as potential tools in cancer therapeutics.

## Author Contributions

WX and YY designed the manuscript. YS and XM wrote and revised the manuscript. MZ searched the references. MW and GW drafted the figures. All authors contributed to the article and approved the submitted version.

## Conflict of Interest

The authors declare that the research was conducted in the absence of any commercial or financial relationships that could be construed as a potential conflict of interest.

## References

[B1] Abdou A. G. Maraee A. H. El-Sayed E. M. Elnaidany N. F. (2011). Immunohistochemical expression of ezrin in cutaneous basal and squamous cell carcinomas. *Ann. Diagn. Pathol.* 15 394–401. 21849257 10.1016/j.anndiagpath.2011.05.005

[B2] Aikawa A. Fujita H. Kosaka T. Minato H. Kiyokawa E. (2019). Clinicopathological significance of heterogeneic ezrin expression in poorly differentiated clusters of colorectal cancers. *Cancer Sci.* 110 2667–2675. 10.1111/cas.14093 31175699 PMC6676292

[B3] Andersson G. Wennersten C. Gaber A. Boman K. Nodin B. Uhlen M. (2014). Reduced expression of ezrin in urothelial bladder cancer signifies more advanced tumours and an impaired survival: validatory study of two independent patient cohorts. *BMC Urol.* 14:36. 10.1186/1471-2490-14-36 24885195 PMC4049499

[B4] Antelmi E. Cardone R. A. Greco M. R. Rubino R. Di Sole F. Martino N. A. (2013). ss1 integrin binding phosphorylates ezrin at T567 to activate a lipid raft signalsome driving invadopodia activity and invasion. *PLoS One* 8:e75113. 10.1371/journal.pone.0075113 24086451 PMC3782503

[B5] Antoine-Bertrand J. Ghogha A. Luangrath V. Bedford F. K. Lamarche-Vane N. (2011). The activation of ezrin-radixin-moesin proteins is regulated by netrin-1 through Src kinase and RhoA/Rho kinase activities and mediates netrin-1-induced axon outgrowth. *Mol. Biol. Cell* 22 3734–3746. 10.1091/mbc.e10-11-0917 21849478 PMC3183026

[B6] Assao A. Nonogaki S. Lauris J. R. P. Carvalho A. L. Pinto C. A. L. Soares F. A. (2017). Podoplanin, ezrin, and Rho-A proteins may have joint participation in tumor invasion of lip cancer. *Clin. Oral. Investig.* 21 1647–1657. 10.1007/s00784-016-1956-3 27628318

[B7] Athanasopoulou A. Aroukatos P. Nakas D. Repanti M. Papadaki H. Bravou V. (2013). Decreased ezrin and paxillin expression in human urothelial bladder tumors correlate with tumor progression. *Urol. Oncol.* 31 836–842. 10.1016/j.urolonc.2011.07.003 21868260

[B8] Autero M. Heiska L. Ronnstrand L. Vaheri A. Gahmberg C. G. Carpen O. (2003). Ezrin is a substrate for Lck in T cells. *FEBS Lett.* 535 82–86. 10.1016/s0014-5793(02)03861-9 12560083

[B9] Auvinen E. Carpen O. Korpela T. Ronty M. Vaheri A. Tarkkanen J. (2013). Altered expression of ezrin, E-cadherin and beta-catenin in cervical neoplasia. *Neoplasma* 60 56–61. 10.4149/neo_2013_008 23067217

[B10] Baumgartner M. Sillman A. L. Blackwood E. M. Srivastava J. Madson N. Schilling J. W. (2006). The Nck-interacting kinase phosphorylates ERM proteins for formation of lamellipodium by growth factors. *Proc. Natl. Acad. Sci. U.S.A.* 103 13391–13396. 10.1073/pnas.0605950103 16938849 PMC1569174

[B11] Bretscher A. Edwards K. Fehon R. G. (2002). ERM proteins and merlin: integrators at the cell cortex. *Nat. Rev. Mol. Cell Biol.* 3 586–599. 10.1038/nrm882 12154370

[B12] Bretscher A. Reczek D. Berryman M. (1997). Ezrin: a protein requiring conformational activation to link microfilaments to the plasma membrane in the assembly of cell surface structures. *J. Cell Sci.* 110(Pt 24), 3011–3018. 9365271 10.1242/jcs.110.24.3011

[B13] Bulut G. Hong S. H. Chen K. Beauchamp E. M. Rahim S. Kosturko G. W. (2012). Small molecule inhibitors of ezrin inhibit the invasive phenotype of osteosarcoma cells. *Oncogene* 31 269–281. 10.1038/onc.2011.245 21706056 PMC3513970

[B14] Cao L. L. Xie J. W. Lin Y. Zheng C. H. Li P. Wang J. B. (2014). miR-183 inhibits invasion of gastric cancer by targeting Ezrin. *Int. J. Clin. Exp. Pathol.* 7 5582–5594. 25337200 PMC4203171

[B15] Cash T. Yin H. McCracken C. Geng Z. DuBois S. G. Shehata B. M. (2017). Correlation of ezrin expression pattern and clinical outcomes in ewing sarcoma. *Sarcoma* 2017:8758623. 10.1155/2017/8758623 PMC5299201 28246524

[B16] Celik H. Bulut G. Han J. Graham G. T. Minas T. Z. Conn E. J. (2016). Ezrin Inhibition up-regulates stress response gene expression. *J. Biol. Chem.* 291 13257–13270. 10.1074/jbc.m116.718189 27137931 PMC4933238

[B17] Celik H. Hong S. H. Colon-Lopez D. D. Han J. Kont Y. S. Minas T. Z. (2015). Identification of novel ezrin inhibitors targeting metastatic osteosarcoma by screening open access malaria box. *Mol. Cancer Ther.* 14 2497–2507. 10.1158/1535-7163.mct-15-0511 26358752 PMC4636458

[B18] Celik H. Sajwan K. P. Selvanathan S. P. Marsh B. J. Pai A. V. Kont Y. S. (2015). Ezrin binds to DEAD-box RNA Helicase DDX3 and regulates its function and protein level. *Mol. Cell. Biol.* 35 3145–3162. 26149384 10.1128/MCB.00332-15 PMC4539380

[B19] Chang Y. T. Peng H. Y. Hu C. M. Huang S. C. Tien S. C. Jeng Y. M. (2020). Pancreatic cancer-derived small extracellular vesical Ezrin regulates macrophage polarization and promotes metastasis. *Am. J. Cancer Res.* 10 12–37. 32064151 PMC7017748

[B20] Chen Q. Y. Xu W. Jiao D. M. Wu L. J. Song J. Yan J. (2013). Silence of ezrin modifies migration and actin cytoskeleton rearrangements and enhances chemosensitivity of lung cancer cells in vitro. *Mol. Cell. Biochem.* 377 207–218. 10.1007/s11010-013-1586-x 23435957

[B21] Chen Q. Y. Yan J. Hu H. Z. Chen F. Y. Song J. Jiang Z. Y. (2012). [Expression of ezrin in human non-small cell lung cancer and its relationship with metastasis and prognosis]. *Zhonghua Zhong Liu Za Zhi* 34 436–440. 22967445 10.3760/cma.j.issn.0253-3766.2012.06.008

[B22] Chen Y. Wang D. Guo Z. Zhao J. Wu B. Deng H. (2011). Rho kinase phosphorylation promotes ezrin-mediated metastasis in hepatocellular carcinoma. *Cancer Res.* 71 1721–1729. 10.1158/0008-5472.can-09-4683 21363921 PMC3119000

[B23] Chetry M. Song Y. Pan C. Li R. Zhang J. Zhu X. (2020). Effects of Galectin-1 on Biological Behavior in Cervical Cancer. *J. Cancer* 11 1584–1595. 10.7150/jca.38538 32047564 PMC6995396

[B24] Cihan Y. B. (2018). Does ezrin play a predictive role in cancer patients undergoing radiotherapy and/or chemotherapy? *Hum. Pathol.* 80 247–248. 10.1016/j.humpath.2018.04.029 29940285

[B25] Clucas J. Valderrama F. (2014). ERM proteins in cancer progression. *J. Cell Sci.* 127(Pt 2), 267–275. 10.1242/jcs.133108 24421310

[B26] Crepaldi T. Gautreau A. Comoglio P. M. Louvard D. Arpin M. (1997). Ezrin is an effector of hepatocyte growth factor-mediated migration and morphogenesis in epithelial cells. *J. Cell Biol.* 138 423–434. 10.1083/jcb.138.2.423 9230083 PMC2138186

[B27] Cui Y. Li T. Zhang D. Han J. (2010). Expression of ezrin and phosphorylated Ezrin (pEzrin) in pancreatic ductal adenocarcinoma. *Cancer Invest* 28 242–247. 10.3109/07357900903124498 20158339

[B28] Denker S. P. Huang D. C. Orlowski J. Furthmayr H. Barber D. L. (2000). Direct binding of the Na–H exchanger NHE1 to ERM proteins regulates the cortical cytoskeleton and cell shape independently of H(+) translocation. *Mol. Cell.* 6 1425–1436. 10.1016/s1097-2765(00)00139-8 11163215

[B29] Di Cristofano C. Leopizzi M. Miraglia A. Sardella B. Moretti V. Ferrara A. (2010). Phosphorylated ezrin is located in the nucleus of the osteosarcoma cell. *Mod. Pathol.* 23 1012–1020. 10.1038/modpathol.2010.77 20348881

[B30] Elliott B. E. Meens J. A. SenGupta S. K. Louvard D. Arpin M. (2005). The membrane cytoskeletal crosslinker ezrin is required for metastasis of breast carcinoma cells. *Breast Cancer Res.* 7 R365–R373. 15987432 10.1186/bcr1006 PMC1143558

[B31] Fadiel A. Choi S. D. Park B. Kim T. H. Buldo-Licciardi J. Ahmadi M. (2017). Expression of ezrin and estrogen receptors during cervical carcinogenesis. *Reprod. Sci.* 24 706–712. 10.1177/1933719116667222 27688241

[B32] Fathi A. Mosaad H. Hussein S. Roshdy M. Ismail E. I. (2017). Prognostic significance of CD133 and ezrin expression in colorectal carcinoma. *IUBMB Life* 69 328–340. 10.1002/iub.1609 28261953

[B33] Federici C. Brambilla D. Lozupone F. Matarrese P. de Milito A. Lugini L. (2009). Pleiotropic function of ezrin in human metastatic melanomas. *Int. J. Cancer* 124 2804–2812. 10.1002/ijc.24255 19235924

[B34] Fehon R. G. McClatchey A. I. Bretscher A. (2010). Organizing the cell cortex: the role of ERM proteins. *Nat. Rev. Mol. Cell Biol.* 11 276–287. 10.1038/nrm2866 20308985 PMC2871950

[B35] Fievet B. T. Gautreau A. Roy C. Del Maestro L. Mangeat P. Louvard D. (2004). Phosphoinositide binding and phosphorylation act sequentially in the activation mechanism of ezrin. *J. Cell Biol.* 164 653–659. 10.1083/jcb.200307032 14993232 PMC2172172

[B36] Fouassier L. Yun C. C. Fitz J. G. Doctor R. B. (2000). Evidence for ezrin-radixin-moesin-binding phosphoprotein 50 (EBP50) self-association through PDZ-PDZ interactions. *J. Biol. Chem.* 275 25039–25045. 10.1074/jbc.c000092200 10859298

[B37] Gary R. Bretscher A. (1995). Ezrin self-association involves binding of an N-terminal domain to a normally masked C-terminal domain that includes the F-actin binding site. *Mol. Biol. Cell* 6 1061–1075. 10.1091/mbc.6.8.1061 7579708 PMC301263

[B38] Gautreau A. Louvard D. Arpin M. (2000). Morphogenic effects of ezrin require a phosphorylation-induced transition from oligomers to monomers at the plasma membrane. *J. Cell Biol.* 150 193–203. 10.1083/jcb.150.1.193 10893267 PMC2185562

[B39] Gautreau A. Poullet P. Louvard D. Arpin M. (1999). Ezrin, a plasma membrane-microfilament linker, signals cell survival through the phosphatidylinositol 3-kinase/Akt pathway. *Proc. Natl. Acad. Sci. U.S.A.* 96 7300–7305. 10.1073/pnas.96.13.7300 10377409 PMC22080

[B40] Gavert N. Ben-Shmuel A. Lemmon V. Brabletz T. Ben-Ze’ev A. (2010). Nuclear factor-kappaB signaling and ezrin are essential for L1-mediated metastasis of colon cancer cells. *J. Cell Sci.* 123(Pt 12), 2135–2143. 10.1242/jcs.069542 20501702 PMC4481617

[B41] Ghaffari A. Hoskin V. Turashvili G. Varma S. Mewburn J. Mullins G. (2019). Intravital imaging reveals systemic ezrin inhibition impedes cancer cell migration and lymph node metastasis in breast cancer. *Breast Cancer Res.* 21:12. 10.1186/s13058-018-1079-7 PMC6345049 30678714

[B42] Gloerich M. ten Klooster J. P. Vliem M. J. Koorman T. Zwartkruis F. J. Clevers H. (2012). Rap2A links intestinal cell polarity to brush border formation. *Nat. Cell Biol.* 14 793–801. 10.1038/ncb2537 22797597

[B43] Haga R. B. Ridley A. J. (2016). Rho GTPases: regulation and roles in cancer cell biology. *Small GTPases* 7 207–221. 10.1080/21541248.2016.1232583 27628050 PMC5129894

[B44] Hao J. J. Liu Y. Kruhlak M. Debell K. E. Rellahan B. L. Shaw S. (2009). Phospholipase C-mediated hydrolysis of PIP2 releases ERM proteins from lymphocyte membrane. *J. Cell Biol.* 184 451–462. 10.1083/jcb.200807047 19204146 PMC2646552

[B45] Hashimoto K. Hayashi R. Mukaigawa T. Yamazaki M. Fujii S. (2017). Concomitant expression of ezrin and HER2 predicts distant metastasis and poor prognosis of patients with salivary gland carcinomas. *Hum. Pathol.* 63 110–119. 10.1016/j.humpath.2017.02.017 28300573

[B46] Hata K. Yoshida J. Udagawa H. Hashimoto H. Fujii S. Hishida T. (2016). The difference in Ezrin-pAkt signaling axis between lepidic and papillary predominant invasive adenocarcinomas of the lung. *J. Cancer Res. Clin. Oncol.* 142 1421–1430. 10.1007/s00432-016-2154-z 27059464 PMC11819307

[B47] Heiska L. Carpen O. (2005). Src phosphorylates ezrin at tyrosine 477 and induces a phosphospecific association between ezrin and a kelch-repeat protein family member. *J. Biol. Chem.* 280 10244–10252. 10.1074/jbc.m411353200 15623525

[B48] Hiscox S. Jiang W. G. (1999). Ezrin regulates cell-cell and cell-matrix adhesion, a possible role with E-cadherin/beta-catenin. *J. Cell Sci.* 112(Pt 18), 3081–3090. 10462524 10.1242/jcs.112.18.3081

[B49] Horwitz V. Davidson B. Stern D. Trope C. G. Tavor Re’em T. Reich R. (2016). Ezrin is associated with disease progression in ovarian carcinoma. *PLoS One* 11:e0162502. 10.1371/journal.pone.0162502 27622508 PMC5021292

[B50] Hoskin V. Ghaffari A. Elliott B. E. (2019). Ezrin, more than a metastatic detERMinant? *Oncotarget* 10 6755–6757. 10.18632/oncotarget.27227 31827718 PMC6887573

[B51] Hu K. Dai H. B. Qiu Z. L. (2016). mTOR signaling in osteosarcoma: oncogenesis and therapeutic aspects (Review). *Oncol. Rep.* 36 1219–1225. 10.3892/or.2016.4922 27430283

[B52] Ilmonen S. Vaheri A. Asko-Seljavaara S. Carpen O. (2005). Ezrin in primary cutaneous melanoma. *Mod. Pathol.* 18 503–510. 10.1038/modpathol.3800300 15475929

[B53] Ivetic A. Ridley A. J. (2004). Ezrin/radixin/moesin proteins and Rho GTPase signalling in leucocytes. *Immunology* 112 165–176. 10.1111/j.1365-2567.2004.01882.x 15147559 PMC1782489

[B54] Janiszewska M. Primi M. C. Izard T. (2020). Cell adhesion in cancer: beyond the migration of single cells. *J. Biol. Chem.* 295 2495–2505. 10.1074/jbc.rev119.007759 31937589 PMC7039572

[B55] Jia M. Yan X. Jiang X. Wu Y. Xu J. Meng Y. (2019). Ezrin, a membrane cytoskeleton cross-linker protein, as a marker of epithelial damage in asthma. *Am. J. Respir. Crit. Care Med.* 199 496–507. 10.1164/rccm.201802-0373oc 30290132 PMC6376623

[B56] Jin J. Jin T. Quan M. Piao Y. Lin Z. (2012). Ezrin overexpression predicts the poor prognosis of gastric adenocarcinoma. *Diagn. Pathol.* 7:135. 10.1186/1746-1596-7-135 23039327 PMC3502371

[B57] Jin T. Jin J. Li X. Zhang S. Choi Y. H. Piao Y. (2014). Prognostic implications of ezrin and phosphorylated ezrin expression in non-small cell lung cancer. *BMC Cancer* 14:191. 10.1186/1471-2407-14-191 24629131 PMC3985600

[B58] Karmakar S. Das C. (2004). Modulation of ezrin and E-cadherin expression by IL-1beta and TGF-beta1 in human trophoblasts. *J. Reprod. Immunol.* 64 9–29. 10.1016/j.jri.2004.04.005 15596224

[B59] Kawaguchi K. Yoshida S. Hatano R. Asano S. (2017). Pathophysiological roles of Ezrin/Radixin/Moesin proteins. *Biol. Pharm. Bull.* 40 381–390. 10.1248/bpb.b16-01011 28381792

[B60] Khanna C. Wan X. Bose S. Cassaday R. Olomu O. Mendoza A. (2004). The membrane-cytoskeleton linker ezrin is necessary for osteosarcoma metastasis. *Nat. Med.* 10 182–186. 10.1038/nm982 14704791

[B61] Kobel M. Langhammer T. Huttelmaier S. Schmitt W. D. Kriese K. Dittmer J. (2006). Ezrin expression is related to poor prognosis in FIGO stage I endometrioid carcinomas. *Mod. Pathol.* 19 581–587. 10.1038/modpathol.3800567 16554733

[B62] Kocher H. M. Sandle J. Mirza T. A. Li N. F. Hart I. R. (2009). Ezrin interacts with cortactin to form podosomal rosettes in pancreatic cancer cells. *Gut* 58 271–284. 10.1136/gut.2008.159871 18852256

[B63] Kolegova E. S. Kakurina G. V. Kostromitskiy D. N. Dobrodeev A. Y. Kondakova I. V. (2020). [Increases in mRNA and protein levels of the genes for the actin-binding proteins profilin, fascin, and ezrin promote metastasis in non-small cell lung cancer]. *Mol. Biol.* 54 285–292. 10.31857/S0026898420020068 32392198

[B64] Kong J. Di C. Piao J. Sun J. Han L. Chen L. (2016). Ezrin contributes to cervical cancer progression through induction of epithelial-mesenchymal transition. *Oncotarget* 7 19631–19642. 10.18632/oncotarget.7779 26933912 PMC4991407

[B65] Kong J. Li Y. Liu S. Jin H. Shang Y. Quan C. (2013). High expression of ezrin predicts poor prognosis in uterine cervical cancer. *BMC Cancer* 13:520. 10.1186/1471-2407-13-520 24182314 PMC4228363

[B66] Krishnan K. Bruce B. Hewitt S. Thomas D. Khanna C. Helman L. J. (2006). Ezrin mediates growth and survival in Ewing’s sarcoma through the AKT/mTOR, but not the MAPK, signaling pathway. *Clin. Exp. Metastasis* 23 227–236. 10.1007/s10585-006-9033-y 17028919

[B67] Lee H. W. Kim E. H. Oh M. H. (2012). Clinicopathologic implication of ezrin expression in non-small cell lung cancer. *Korean J. Pathol.* 46 470–477. 10.4132/koreanjpathol.2012.46.5.470 23136574 PMC3490123

[B68] Leiphrakpam P. D. Rajput A. Mathiesen M. Agarwal E. Lazenby A. J. Are C. (2014). Ezrin expression and cell survival regulation in colorectal cancer. *Cell. Signal.* 26 868–879. 10.1016/j.cellsig.2014.01.014 24462708 PMC3974425

[B69] Li J. Wei K. Yu H. Jin D. Wang G. Yu B. (2015). Prognostic value of ezrin in various cancers: a systematic review and updated meta-analysis. *Sci. Rep.* 5:17903. 10.1038/srep17903 PMC4668575 26632332

[B70] Li L. Wang Y. Y. Zhao Z. S. Ma J. (2011). Ezrin is associated with gastric cancer progression and prognosis. *Pathol. Oncol. Res.* 17 909–915. 10.1007/s12253-011-9402-y 21717114

[B71] Li M. Feng Y. M. Fang S. Q. (2017). Overexpression of ezrin and galectin-3 as predictors of poor prognosis of cervical cancer. *Braz. J. Med. Biol. Res.* 50:e5356. 10.1590/1414-431X20165356 PMC5423742 28355349

[B72] Li N. Kong J. Lin Z. Yang Y. Jin T. Xu M. (2019). Ezrin promotes breast cancer progression by modulating AKT signals. *Br. J. Cancer* 120 703–713. 10.1038/s41416-019-0383-z 30804430 PMC6461860

[B73] Li Q. Gao H. Xu H. Wang X. Pan Y. Hao F. (2012). Expression of ezrin correlates with malignant phenotype of lung cancer, and in vitro knockdown of ezrin reverses the aggressive biological behavior of lung cancer cells. *Tumour. Biol.* 33 1493–1504. 10.1007/s13277-012-0400-9 22528947

[B74] Liang F. Wang Y. Shi L. Zhang J. (2017). Association of ezrin expression with the progression and prognosis of gastrointestinal cancer: a meta-analysis. *Oncotarget* 8 93186–93195. 10.18632/oncotarget.21473 29190988 PMC5696254

[B75] Lin L. J. Chen L. T. (2013). Association between ezrin protein expression and the prognosis of colorectal adenocarcinoma. *Mol. Med. Rep.* 8 61–66. 10.3892/mmr.2013.1490 23708420

[B76] Liu Y. Chen Y. Wang X. Zhao P. Zhu Y. Qi Z. (2020). Ezrin is essential for the entry of Japanese encephalitis virus into the human brain microvascular endothelial cells. *Emerg Microbes Infect* 9 1330–1341. 10.1080/22221751.2020.1757388 32538298 PMC7473060

[B77] Lowery A. J. Miller N. Dwyer R. M. Kerin M. J. (2010). Dysregulated miR-183 inhibits migration in breast cancer cells. *BMC Cancer* 10:502. 10.1186/1471-2407-10-502 20858276 PMC2955037

[B78] Mak H. Naba A. Varma S. Schick C. Day A. SenGupta S. K. (2012). Ezrin phosphorylation on tyrosine 477 regulates invasion and metastasis of breast cancer cells. *BMC Cancer* 12:82. 10.1186/1471-2407-12-82 22397367 PMC3372425

[B79] Malmstrom P. U. Hemdan T. Segersten U. (2017). Validation of the ezrin, CK20, and Ki-67 as potential predictive markers for BCG instillation therapy of non-muscle-invasive bladder cancer. *Urol. Oncol.* 35 532.e1–532.e6. 10.1016/j.urolonc.2017.03.010 28389159

[B80] Matsui T. Maeda M. Doi Y. Yonemura S. Amano M. Kaibuchi K. (1998). Rho-kinase phosphorylates COOH-terminal threonines of ezrin/radixin/moesin (ERM) proteins and regulates their head-to-tail association. *J. Cell Biol.* 140 647–657. 10.1083/jcb.140.3.647 9456324 PMC2140160

[B81] McClatchey A. I. (2003). Merlin and ERM proteins: unappreciated roles in cancer development? *Nat. Rev. Cancer* 3 877–883. 10.1038/nrc1213 14668818

[B82] Meng Y. Lu Z. Yu S. Zhang Q. Ma Y. Chen J. (2010). Ezrin promotes invasion and metastasis of pancreatic cancer cells. *J. Transl. Med.* 8:61. 10.1186/1479-5876-8-61 20569470 PMC2916894

[B83] Michie K. A. Bermeister A. Robertson N. O. Goodchild S. C. Curmi P. M. G. (2019). Two sides of the coin: Ezrin/Radixin/Moesin and merlin control membrane structure and contact inhibition. *Int. J. Mol. Sci.* 20:1996. 10.3390/ijms20081996 31018575 PMC6515277

[B84] Moodley S. Lian E. Y. Crupi M. J. F. Hyndman B. D. Mulligan L. M. (2020). RET isoform-specific interaction with scaffold protein Ezrin promotes cell migration and chemotaxis in lung adenocarcinoma. *Lung Cancer* 142 123–131. 10.1016/j.lungcan.2020.02.004 32146264

[B85] Morales F. C. Molina J. R. Hayashi Y. Georgescu M. M. (2010). Overexpression of ezrin inactivates NF2 tumor suppressor in glioblastoma. *Neuro. Oncol.* 12 528–539. 10.1093/neuonc/nop060 20156804 PMC2940645

[B86] Nakamura N. Oshiro N. Fukata Y. Amano M. Fukata M. Kuroda S. (2000). Phosphorylation of ERM proteins at filopodia induced by Cdc42. *Genes Cells* 5 571–581. 10.1046/j.1365-2443.2000.00348.x 10947843

[B87] Okamura D. Ohtsuka M. Kimura F. Shimizu H. Yoshidome H. Kato A. (2008). Ezrin expression is associated with hepatocellular carcinoma possibly derived from progenitor cells and early recurrence after surgical resection. *Mod. Pathol.* 21 847–855. 10.1038/modpathol.2008.59 18425081

[B88] Osawa H. Smith C. A. Ra Y. S. Kongkham P. Rutka J. T. (2009). The role of the membrane cytoskeleton cross-linker ezrin in medulloblastoma cells. *Neuro. Oncol* 11 381–393. 10.1215/15228517-2008-110 19088174 PMC2743218

[B89] Paige M. Kosturko G. Bulut G. Miessau M. Rahim S. Toretsky J. A. (2014). Design, synthesis and biological evaluation of ezrin inhibitors targeting metastatic osteosarcoma. *Bioorg. Med. Chem.* 22 478–487. 10.1016/j.bmc.2013.11.003 24326277 PMC4349528

[B90] Palou J. Algaba F. Vera I. Rodriguez O. Villavicencio H. Sanchez-Carbayo M. (2009). Protein expression patterns of ezrin are predictors of progression in T1G3 bladder tumours treated with nonmaintenance bacillus calmette-guerin. *Eur. Urol.* 56 829–836. 10.1016/j.eururo.2008.09.062 18926620

[B91] Patara M. Santos E. M. Coudry Rde A. Soares F. A. Ferreira F. O. Rossi B. M. (2011). Ezrin expression as a prognostic marker in colorectal adenocarcinoma. *Pathol. Oncol. Res.* 17 827–833. 10.1007/s12253-011-9389-4 21465252

[B92] Pore D. Bodo J. Danda A. Yan D. Phillips J. G. Lindner D. (2015). Identification of Ezrin-Radixin-Moesin proteins as novel regulators of pathogenic B-cell receptor signaling and tumor growth in diffuse large B-cell lymphoma. *Leukemia* 29 1857–1867. 10.1038/leu.2015.86 25801911 PMC4558318

[B93] Poullet P. Gautreau A. Kadare G. Girault J. A. Louvard D. Arpin M. (2001). Ezrin interacts with focal adhesion kinase and induces its activation independently of cell-matrix adhesion. *J. Biol. Chem.* 276 37686–37691. 10.1074/jbc.m106175200 11468295

[B94] Prag S. Parsons M. Keppler M. D. Ameer-Beg S. M. Barber P. Hunt J. (2007). Activated ezrin promotes cell migration through recruitment of the GEF Dbl to lipid rafts and preferential downstream activation of Cdc42. *Mol. Biol. Cell* 18 2935–2948. 10.1091/mbc.e06-11-1031 17538024 PMC1949366

[B95] Quan C. Sun J. Lin Z. Jin T. Dong B. Meng Z. (2019). Ezrin promotes pancreatic cancer cell proliferation and invasion through activating the Akt/mTOR pathway and inducing YAP translocation. *Cancer Manag. Res.* 11 6553–6566. 10.2147/cmar.s202342 31372056 PMC6634270

[B96] Ren L. Khanna C. (2014). Role of ezrin in osteosarcoma metastasis. *Adv. Exp. Med. Biol.* 804 181–201. 10.1007/978-3-319-04843-7_10 24924175

[B97] Riecken L. B. Zoch A. Wiehl U. Reichert S. Scholl I. Cui Y. (2016). CPI-17 drives oncogenic Ras signaling in human melanomas via Ezrin-Radixin-Moesin family proteins. *Oncotarget* 7 78242–78254. 10.18632/oncotarget.12919 27793041 PMC5346635

[B98] Roy C. Martin M. Mangeat P. (1997). A dual involvement of the amino-terminal domain of ezrin in F- and G-actin binding. *J. Biol. Chem.* 272 20088–20095. 10.1074/jbc.272.32.20088 9242682

[B99] Saavedra K. P. Brebi P. M. Roa J. C. (2012). Epigenetic alterations in preneoplastic and neoplastic lesions of the cervix. *Clin. Epigenetics* 4:13. 10.1186/1868-7083-4-13 22938091 PMC3502457

[B100] Saito S. Yamamoto H. Mukaisho K. Sato S. Higo T. Hattori T. (2013). Mechanisms underlying cancer progression caused by ezrin overexpression in tongue squamous cell carcinoma. *PLoS One* 8:e54881. 10.1371/journal.pone.0054881 23357878 PMC3554659

[B101] Saygideger-Kont Y. Minas T. Z. Jones H. Hour S. Celik H. Temel I. (2016). Ezrin enhances EGFR signaling and modulates erlotinib sensitivity in non-small cell lung cancer cells. *Neoplasia* 18 111–120. 10.1016/j.neo.2016.01.002 26936397 PMC5005263

[B102] Shiue H. Musch M. W. Wang Y. Chang E. B. Turner J. R. (2005). Akt2 phosphorylates ezrin to trigger NHE3 translocation and activation. *J. Biol. Chem.* 280 1688–1695. 10.1074/jbc.m409471200 15531580 PMC1237052

[B103] Slik K. Kurki S. Korpela T. Carpen O. Korkeila E. Sundstrom J. (2017). Ezrin expression combined with MSI status in prognostication of stage II colorectal cancer. *PLoS One* 12:e0185436. 10.1371/journal.pone.0185436 28953975 PMC5617236

[B104] Srivastava J. Elliott B. E. Louvard D. Arpin M. (2005). Src-dependent ezrin phosphorylation in adhesion-mediated signaling. *Mol. Biol. Cell* 16 1481–1490. 10.1091/mbc.e04-08-0721 15647376 PMC551509

[B105] Sun N. Wang C. Y. Sun Y. Q. Ruan Y. J. Huang Y. Y. Su T. (2018). Down-regulated miR-148b increases resistance to CHOP in diffuse large B-cell lymphoma cells by rescuing Ezrin. *Biomed. Pharmacother.* 106 267–274. 10.1016/j.biopha.2018.06.093 29966970

[B106] Tang F. Zou F. Peng Z. Huang D. Wu Y. Chen Y. (2011). N,N’-dinitrosopiperazine-mediated ezrin protein phosphorylation via activation of Rho kinase and protein kinase C is involved in metastasis of nasopharyngeal carcinoma 6-10B cells. *J. Biol. Chem.* 286 36956–36967. 10.1074/jbc.m111.259234 21878630 PMC3196127

[B107] Tuna B. Yorukoglu K. Mazzucchelli R. Mungan U. Secil M. Montironi R. (2009). Ezrin immunoreactivity in renal cell carcinomas. *Anal. Quant. Cytol. Histol.* 31 340–344. 20701102

[B108] Viswanatha R. Ohouo P. Y. Smolka M. B. Bretscher A. (2012). Local phosphocycling mediated by LOK/SLK restricts ezrin function to the apical aspect of epithelial cells. *J. Cell Biol.* 199 969–984. 10.1083/jcb.201207047 23209304 PMC3518218

[B109] Wald F. A. Oriolo A. S. Mashukova A. Fregien N. L. Langshaw A. H. Salas P. J. (2008). Atypical protein kinase C (iota) activates ezrin in the apical domain of intestinal epithelial cells. *J. Cell Sci.* 121(Pt 5), 644–654. 10.1242/jcs.016246 18270268 PMC2293289

[B110] Wang G. Mao W. Zheng S. (2008). MicroRNA-183 regulates Ezrin expression in lung cancer cells. *FEBS Lett.* 582 3663–3668. 10.1016/j.febslet.2008.09.051 18840437

[B111] Wu B. Li J. Huang D. Wang W. Chen Y. Liao Y. (2011). Baicalein mediates inhibition of migration and invasiveness of skin carcinoma through Ezrin in A431 cells. *BMC Cancer* 11:527. 10.1186/1471-2407-11-527 22204275 PMC3260329

[B112] Yang H. S. Hinds P. W. (2003). Increased ezrin expression and activation by CDK5 coincident with acquisition of the senescent phenotype. *Mol. Cell.* 11 1163–1176. 10.1016/s1097-2765(03)00135-7 12769842

[B113] Yang H. S. Hinds P. W. (2006). Phosphorylation of ezrin by cyclin-dependent kinase 5 induces the release of Rho GDP dissociation inhibitor to inhibit Rac1 activity in senescent cells. *Cancer Res.* 66 2708–2715. 10.1158/0008-5472.can-05-3141 16510591

[B114] Yeh C. N. Pang S. T. Chen T. W. Wu R. C. Weng W. H. Chen M. F. (2009). Expression of ezrin is associated with invasion and dedifferentiation of hepatitis B related hepatocellular carcinoma. *BMC Cancer* 9:233. 10.1186/1471-2407-9-233 19604375 PMC2716370

[B115] Yin L. M. Duan T. T. Ulloa L. Yang Y. Q. (2018). Ezrin orchestrates signal transduction in airway cells. *Rev. Physiol. Biochem. Pharmacol.* 174 1–23. 10.1007/112_2017_4 28702704 PMC6289583

[B116] Yu N. Fu S. Liu Y. Xu Z. Hao J. Wang B. (2015). miR-96 suppresses renal cell carcinoma invasion via downregulation of Ezrin expression. *J. Exp. Clin. Cancer Res.* 34:107. 10.1186/s13046-015-0224-8 PMC4588898 26419932

[B117] Zacapala-Gomez A. E. Navarro-Tito N. Alarcon-Romero L. D. C. Ortuno-Pineda C. Illades-Aguiar B. Castaneda-Saucedo E. (2018). Ezrin and E-cadherin expression profile in cervical cytology: a prognostic marker for tumor progression in cervical cancer. *BMC Cancer* 18:349. 10.1186/s12885-018-4243-7 29587669 PMC5872531

[B118] Zhang X. Li G. Guo Y. Song Y. Chen L. Ruan Q. (2019). Regulation of ezrin tension by S-nitrosylation mediates non-small cell lung cancer invasion and metastasis. *Theranostics* 9 2555–2571. 10.7150/thno.32479 31131053 PMC6525990

[B119] Zhang X. Q. Chen G. P. Wu T. Yan J. P. Zhou J. Y. (2012). Expression and clinical significance of ezrin in non–small-cell lung cancer. *Clin. Lung Cancer* 13 196–204. 10.1016/j.cllc.2011.04.002 22137559

[B120] Zhang Y. Zhang L. Zhang G. Li S. Duan J. Cheng J. (2014). Osteosarcoma metastasis: prospective role of ezrin. *Tumour. Biol.* 35 5055–5059. 10.1007/s13277-014-1799-y 24609902

